# Trends in the association between educational assortative mating, infant and child mortality in Nigeria

**DOI:** 10.1186/s12889-021-11568-0

**Published:** 2021-08-03

**Authors:** Tolulope Ariyo, Quanbao Jiang

**Affiliations:** Institute for Population and Development Studies, School of Public Policy and Administration, Xi’an Jiaotong University, Xi’an, China

**Keywords:** Educational homogamy, Parent education, Assortative matching, Childhood survival, Under-five mortality, Sub-Sahara Africa

## Abstract

**Background:**

Existing knowledge has established the connection between maternal education and child survival, but little is known about how educational assortative mating (EAM), relates to childhood mortality. We attempt to examine this association in the context of Nigeria.

**Methods:**

Data was obtained from the 2008, 2013, and 2018 waves of the Nigeria Demographic and Health Survey, which is a cross-sectional study. The sample includes the analysis of 72,527 newborns within the 5 years preceding each survey. The dependent variables include the risk of a newborn dying before 12 months of age (infant mortality), or between the age of 12–59 months (child mortality). From the perspective of the mother, the independent variable, EAM, includes four categories (high-education homogamy, low-education homogamy, hypergamy, and hypogamy). The Cox proportional hazard regression was employed for multivariate analyses, while the estimation of mortality rates across the spectrum of EAM was obtained through the synthetic cohort technique.

**Results:**

The risk of childhood mortality varied across the spectrum of EAM and was particularly lowest among those with high-education homogamy. Compared to children of mothers in low-education homogamy, children of mothers in high-education homogamy had 25, 31 to 19% significantly less likelihood of infant mortality, and 34, 41, and 57% significantly less likelihood of child mortality in 2008, 2013 and 2018 survey data, respectively. Also, compared to children of mothers in hypergamy, children of mothers in hypogamous unions had 20, 12, and 11% less likelihood of infant mortality, and 27, 36, and 1% less likelihood of child mortality across 2008, 2013 and 2018 surveys, respectively, although not significant at *p* < 0.05. Both infant and child mortality rates were highest in low-education homogamy, as expected, lowest in high-education homogamy, and lower in hypogamy than in hypergamy. Furthermore, the trends in the rate declined between 2008 and 2018, and were higher in 2018 than in 2013.

**Conclusion:**

This indicates that, beyond the absolute level of education, the similarities or dissimilarities in partners’ education may have consequences for child survival, alluding to the family system theory. Future studies could investigate how this association varies when marital status is put into consideration.

**Supplementary Information:**

The online version contains supplementary material available at 10.1186/s12889-021-11568-0.

## Background

The indicators of mortality, including infant and child mortality, are important indices for assessing community health status and human capital development [1, 2]. Infant mortality refers to the probability of dying within 11 months of birth (age 0–11 months), while child mortality is the probability of dying before the fifth birthday, having survived until the first birthday (age 12–59 months) [3]. Although both infant and child mortality rates in Nigeria, as well as in the sub-Sahara Africa (SSA) region, have been steadily declining for decades, they are still relatively high compared to other developed countries [4].

A large body of literature has reported that increased maternal education offers protection from childhood mortality [5, 6]. Even though only a few studies have investigated the same for paternal education, some have also reported a similar relationship [7, 8]. However, educational assortative mating (EAM), which defines relationships based on homogeneity or differences in a couple’s educational attainment, has received little attention in terms of the implications for child health outcomes. A woman could have the same level of education as her partner (homogamy), less education (hypergamy) or more education, (hypogamy), and each of them may have a differing result in child health outcomes.

The theoretical basis of the association between EAM and child survival is engrained in the family system theory. Family system theory suggests that families operate as a unit, with complex interaction and independencies [9–11]. According to Rauscher (2020), connections between family system theory and EAM may be explained from three perspectives, including stress/support, parenting practices, and pooled resources [12]. First, similarities in educational attainment could translate into better understanding and interactions between spouses [13], culminating in both emotional and instrumental support during prenatal stages, which may relate to better pregnancy outcomes [14, 15] or improve infant and childcare [16]. Dissimilarities in educational attainment, on the other hand, may be associated with frequent friction [13], causing both emotional, mental, or physical stress on the part of the mother during the prenatal stage [17], or resulting in childcare neglect [18].

Secondly, parents’ educational similarities could encourage better consensus and consistency in prenatal healthcare choices and behavior, including abstaining from unhealthy practices that have implications for infant health outcomes during the prenatal stages [19]. Also, it could help with better management of morbidities [20], or even good nutritional practices as an approach to healthy living. All of which are important for better health outcomes for infants and children.

Thirdly, according to the Andersen behavioral model of healthcare utilization, financial resources could act as an enabling factor in healthcare utilization [21]. As educational attainment may relate to income, EAM could also be related to family wealth [22]. Couples may be able to pool resources together, providing better access to healthcare which may translate into better child health outcomes. This phenomenon is likely to be germane in low- and middle-income economies where policies such as medical insurance or support are non-statutory provisions.

Studies from high-income countries concerning the nexus between EAM and child health outcomes are limited. However, evidence from the United States suggests that parents’ educational homogamy is beneficial for child health while hypergamy is detrimental [12]. In low- and middle-income countries, evidence from a study with samples cutting across South America (Peru), Asia (India and Vietnam) and Sub-Sahara Africa (Ethiopia) suggests that educational homogamy is positively associated with indicators of child stunting and thinness in Peru and Vietnam, but negatively associated in India and Ethiopia [13]. Another cross-country study across 21 African countries reported that EAM does not significantly predict under-5 mortality in 18 countries, including Nigeria [23].

When under-5 mortality is decomposed, the findings from the 21-cross-country study may not be a complete reflection of infant mortality in particular. While morbidities of preventable diseases such as malaria, diarrhea, chronic malnutrition, and so on are common causes of child deaths [24, 25], deaths within the first 11 months of life (including neonates) have been linked to preterm births, short birth intervals, fertility behavior, household wealth, and environmental factors among other things [26–30]. Many of these factors concern decisions relating to health, fertility, or the household. Decision-making strategies might defer across homogamous, hypergamous, or hypogamous unions, thereby causing the magnitude of any potential association to vary across the spectrum of EAM. As a result, to the extent that the association between EAM and childhood mortality is not fully established in the context of Nigeria, our study aims to examine this in light of infant and child mortality, as well as provide trends from three waves of data.

## Methods

### Setting and data

This study analyzed data obtained from three waves of the Nigeria Demographic and Health Survey (DHS). It includes the surveys of 2008, 2013, and 2018, collected from June to October 2008, February to July 2013, and August to December 2018, respectively. Nigeria is located in West Africa, and the survey was conducted by the National Population Commission (NPC) in collaboration with ICF International. The study protocols followed the Helsinki guidelines, and also received approval from the National Health Research Ethics Committee of Nigeria. The sample for the survey was selected using a stratified two-stage cluster design. This consisted of the selection of primary sampling units (PSUs) across both rural and urban strata. A nationally representative sample of 36,269; 40,680; and 41,668 households were selected across the PSUs in 2008, 2013 and 2018, respectively. All women aged 15 to 49 years old in households were eligible for interviews. Out of the 34,596; 39,902; and 42,121 eligible women in 2008, 2013 and 2018, respectively, between 97 to 99% were successfully interviewed in each of the years. Questions related to household socio-demography, reproductive health, and fertility, among others. No financial compensation was attached to the respondent’s participation. More information about the survey setting and data collection is available in the final reports [31–33].

### Sample selection

Sample selection focused on children who had complete information about their survival status and the educational attainment of both parents. Children of mothers who are either separated, widowed, not cohabitating, or not in a marital union were excluded from the analysis. This was done because the direct or indirect input of the father into childcare could be limited or non-existent (as would be in the case of widowed mothers or single mothers not in a marital union). In addition, mothers who have been married more than once were also excluded because elements from the previous union(s) may have unexplained residuals in the current union. This reduced the sample to a total of 72,527 out of 94,053 births across the three surveys. Analysis of infant mortality included all 72,527 children (2008 = 21,716; 2013 = 23,981; 2018 = 26,930), and child mortality included 53,595 children (2008 = 15,809; 2013 = 17,758; 2018 = 20,028). However, analysis varied slightly across models, depending on the number of missing values among covariates, varying up to 5.3%.

### Variables and measures

#### Outcome variables

The outcome variables in this study were the risk of infant and child mortality. During the survey, the women were asked about the number of births they had in the 5 years preceding the date of the survey, and the survival status of the child. Where a child was reported dead, the age at death was inquired. We combined the children’s survival status and the current age, or age at death, to generate the outcome variables for the survival analysis. Children who had died (i.e. non-censored) were regarded as cases, whereas children who were still alive at the time of the survey were treated as right-censored.

#### Independent variable

The independent variable in this study was EAM, generated from information on the level of education of both the mother and the father. During the survey, the mother responded to questions about her highest level of educational attainment, as well as that of her partner. For both the mother and the partner, the responses were in four variants, (1) no education, (2) primary education, (3) secondary education, and (4) tertiary education.

Although, most studies on EAM have used a single construct of the homogamy category. However, given that child health outcomes are strongly associated with parents’ level of educational attainment, we sub-divided homogamy into low and high education. Relative to the mother, we generated four categories. (1) Low-education homogamy–both parents have at most primary school education, (2) High-education homogamy–both parents have at least secondary education, (3) Hypergamy –the father has at least secondary and the mother has at most primary, (4) Hypogamy – the father has at most primary and the mother has at least secondary education. This pattern follows the perception that people who have primary education and no education tend to have similar child health outcomes, while those having secondary education and above equally share similar child health outcomes [5, 34]. For a robust comparison, low-education homogamy was taken as a reference for all other categories and hypergamy was again taken separately as a reference for hypogamy.

#### Covariates

Several variables at the child, maternal, household, and community levels which have been described as possible determinants of infant and child mortality were included as covariates [26–29, 35]. Child-level variables include *gender, birth order*, *birth interval*, *perceived birth weight*, *type of birth* (singleton vs. multiple births), and the *place of delivery* (medical institution vs. non-medical institution).

Maternal-related variables include *chronological age,* the *number of surviving children,* and e*mployment status.* Household-related variables include the *source of drinking water, distance to a medical facility (a* problem vs. not a problem*), religion, and family wealth.* The family wealth index was derived through principal component analysis based on the number and kinds of assets each household owns, ranging from a television to a bicycle or car, and housing characteristics such as toilet facilities and flooring materials. The index was already available as part of the Nigeria DHS dataset. Community-level covariates include the *geopolitical zone* of residency and *rural/urban* residency.

### Analysis

For multivariate analysis, we used the Cox proportional hazard regression with the frailty model. This was done for two reasons. First, Cox regression analysis allows for the inclusion of censored data and it models censored time-until-event data as a dependent variable. Secondly, the frailty model considers the hierarchical structure of the data. Children are nested within households, and, as such, have shared frailty, which may make subjects within households correlate [36].

Conditioned on frailty *α*_*i*_, the hazard function *h*_*ij*_(*t*) for the failure time of the j^th^ child in the i^th^ household (j = 1, 2, 3, … ..j; i = 1, 2, 3 … ..n) is expressed as

1 $$ {h}_{ij}(t)={h}_0(t){\alpha}_i\mathit{\exp}\left({x}_{ij}\beta \right) $$

Where *h*_0_(*t*) is an unspecified baseline hazard function, *α*_*i*_ is the group-level (household) frailty and *β*_*x*_ denotes the vector of the regression coefficient for a set of covariates [37, 38].

The analyses include two models. Model 1 included the dependent variable and covariates, and Model 2 included an interaction between EAM and household wealth. This is based on the Andersen behavioral model of health service utilization [21], as family wealth may affect factors such as access to quality health and nutrition, which are among the determinants of childhood mortality [26–29].

### Sensitivity analysis

We conducted three sensitivity analyses to check the robustness of our result. In the first, we restructured the EAM variable by re-segmenting it into at most secondary vs. tertiary. In the second, we excluded couples who have the same level of educational attainment and then defined homogamy as a mother having any level of education higher than her husband’s, and hypergamy as a father having any level of education higher than the mother. In the third, we excluded those with no years of formal education and then created an indicator for whether the number of years of education completed by the mother is lower, equal, or higher than the number of years completed by the father.

**Fig. 1:**
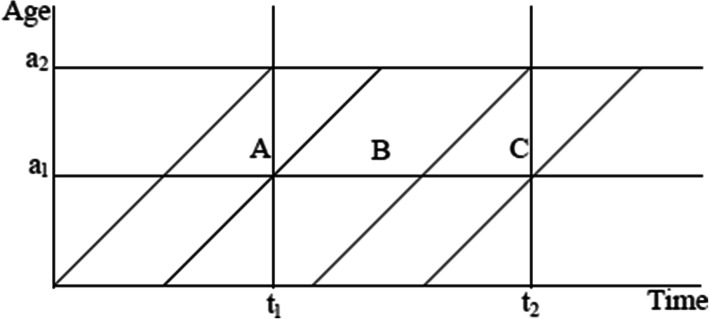
Cohorts used to calculate synthetic rates

The synthetic cohort technique, as modified by Rutstein (1983) [39], was employed to estimate infant and child mortality rates across the spectrum of EAM. The probability of dying is built up from probabilities calculated for subintervals: 0, 1–2, 3–5, 6–11 months (1q_0_), and 12–23, 24–35, 36–47, 48–59 months (4q_1_). Death probabilities are defined by a time-period and an age interval, and within these two parameters, three birth cohorts of children are included, as illustrated in Fig. 1.

Only cohort B is completely included, while cohorts A and C are only partially exposed to mortality in the time-period [t_1_ t_2_]. The subinterval probabilities of death are calculated by dividing the number of deaths occurring in the relevant age interval for children who were exposed to death in a specific calendar period by the number of children exposed in the calendar period. See the DHS statistics guide for a more detailed explanation of this methodology [40, 41]. The conventional mortality rate is then given as:

2 $$ n{\mathrm{q}}_x=\left(1-\prod \limits_{i=x}^{i=x+n}\left(1-q\left[i\right]\right)\right)\times 1000 $$

where *n*q_*x*_ is the probability of dying between ages *x* and *x* + *n* , and *q*[*i*] is quotient from the subinterval probabilities of dying.

The strength of the approach is rooted in the fact that it allows the full use of the most recent data.

The procedure was implemented using the command “*syncmrate*” in STATA. All analyses were done using STATA software version 13.0 (StataCorp, College Station, TX, USA), and results were reported at a 95% significance threshold.

## Results

### Summary of sample characteristics

The socio-demographic characteristics of the pooled sample are presented in Table 1. The gender of the children born within the 5 years preceding each wave of the survey was a nearly equal distribution (51.0% male, and 49.0% female). The majority of the births (64.1%) were delivered at a medical facility, and 96.6% were singletons. The average age of mothers was 29.3 years, standard deviation (SD = 6.8). Furthermore, about 68.2% of households were from rural areas, and a negative average family wealth index of − 0.2 (SD = 1.0) suggests that the majority of the sample were from poor households.

**Table 1:** Percentage of the distribution of the pooled sample characteristics *N* = 72,527: 2008–2018 Nigeria DHS

Variable	n	%	Variable	n	%	Mean	SD
Gender			Mother’s age (in years)	72,527		29.3	6.8
Male	36,962	51.0	No. of living children	72,527		3.7	2.1
Female	35,565	49.0	Mother’s employment †	**72,462**			
Birth order			Not employed	24,248	33.5		
Firstborn	13,292	18.3	employed	48,214	66.5		
2-3rd	24,431	33.7	Source of drinking water †	**72,082**			
4th +	34,804	48.0	Unimproved	41,312	57.3		
Birth interval ^a^	**72,643**		Improved	30,770	42.7		
< 24 months	14,234	19.7	Distance to medical facility †	**72,348**			
> 24 months	58,409	80.3	A big problem	24,649	34.1		
Perceived birth weight ^a^	**71,100**		Not a problem	47,699	65.9		
Small	10,249	14.4	Household wealth index	72,527		−0.2	1.0
Average	31,638	44.5	Religion †	**72,258**			
Large	29,213	41.1	Muslim	27,306	37.8		
Type of Birth			Christian	44,047	61.0		
Singleton	70,025	96.6	Others	905	1.2		
Multiple births	2502	3.4	Geopolitical zone				
Place of delivery ^a^	**72,065**		Northcentral	12,322	17.0		
Non-medical	46,208	64.1	Northeast	15,936	22.0		
Medical	25,857	35.9	Northwest	22,515	31.0		
			Southeast	6647	9.2		
			Southsouth	7101	9.8		
			Southwest	8006	11.0		
			Metropolitan status				
			Urban	23,041	31.8		
			Rural	49,486	68.2		

^a^ *N* < 72,527

### Multivariate analysis

The main assumption of the Cox regression is proportional hazard, and we used the scaled Schoenfeld residuals test to verify that this was not violated [36]. The test of proportionality of the whole, as well as each predictor and covariates, were not significant (*p*-values > 0.05). Also, the graphed plot of the log(−log (survival)) of the main predictor variable against survival time showed roughly parallel lines for all categories of EAM (particularly after the neonate period), an indication of proportional hazard (see supplemental Figure 1).

#### The relationship between EAM and infant mortality

The result of the unadjusted model is shown in supplemental Table 1 and it indicates some significant relationships between EAM, infant, and child mortality across the three surveys. Based on the adjusted result of the 2018 survey, compared to the children of mothers in low-education homogamy, those of mothers in high-education homogamy had 19% (HR = 0.81; CI = 0.69–0.97; *p* < 0.05) less likelihood of infant mortality (see Table 2, Model 1). Also, compared to children of mothers in low-education homogamy, the likelihood of infant mortality was lessened by 1% (HR = 0.99; CI = 0.85–1.16; *p* > 0.1) for children of mothers in hypergamous union and by 11% (HR = 0.89; CI = 0.69–1.13; *p* > 0.1) for children of mothers in hypogamous union, although these were not significant at *p* < 0.05 (see Table 2, Model 1).

**Table 2:** Cox proportional regression showing the adjusted hazard ratio between educational assortative mating, infant mortality: 2008–2018 Nigeria DHS

VARIABLES	2008				2013				2018			
	Mode 1		Model 2		Model 1		Model 2		Model 1		Model 2	
	HR	CI	HR	CI	HR	CI	HR	CI	HR	CI	HR	CI
EAM												
Homogamy low (ref)												
Homogamy high	0.75**	(0.61–0.93)	0.78*	(0.64–0.95)	0.69***	(0.56–0.85)	0.71***	(0.58–0.87)	0.81*	(0.67–0.97)	0.84	(0.70–1.01)
Hypergamy	1.08	(0.92–1.26)	0.97	(0.82–1.14)	0.96	(0.81–1.13)	0.91	(0.76–1.09)	0.99	(0.85–1.16)	0.99	(0.83–1.19)
Hypogamy	0.87	(0.67–1.12)	0.80	(0.62–1.03)	0.84	(0.65–1.09)	0.80	(0.61–1.05)	0.89	(0.69–1.13)	0.85	(0.65–1.10)
Female (ref = male)	0.79***	(0.72–0.88)	0.80***	(0.72–0.88)	0.85**	(0.76–0.94)	0.85**	(0.76–0.94)	0.85**	(0.77–0.94)	0.85**	(0.77–0.94)
Birth order												
Firstborn (ref)												
2-4th	0.95	(0.81–1.12)	0.95	(0.81–1.12)	0.93	(0.79–1.11)	0.93	(0.79–1.10)	0.87	(0.74–1.02)	0.86	(0.73–1.01)
5th+	2.19***	(1.79–2.68)	2.18***	(1.78–2.66)	2.34***	(1.91–2.87)	2.33***	(1.90–2.85)	2.32***	(1.90–2.82)	2.29***	(1.88–2.79)
Birth interval												
< 24 months (ref)												
> 24 months	0.53***	(0.47–0.60)	0.53***	(0.47–0.60)	0.57***	(0.50–0.65)	0.57***	(0.50–0.65)	0.55***	(0.49–0.61)	0.55***	(0.49–0.61)
Perceived birth weight												
Small (ref)												
Average	0.72***	(0.62–0.83)	0.71***	(0.62–0.82)	0.85*	(0.74–0.99)	0.85*	(0.74–0.99)	0.71***	(0.62–0.80)	0.71***	(0.62–0.80)
Large	0.68***	(0.59–0.79)	0.68***	(0.59–0.78)	0.77***	(0.67–0.90)	0.77***	(0.67–0.90)	0.71***	(0.61–0.81)	0.71***	(0.62–0.82)
Multiple births (ref = singleton)	3.81***	(3.07–4.73)	3.81***	(3.07–4.73)	3.89***	(3.16–4.79)	3.86***	(3.14–4.76)	4.01***	(3.39–4.74)	3.98***	(3.36–4.71)
Place of delivery												
Medical (ref non-medical)	1.11	(0.95–1.31)	1.11	(0.95–1.30)	0.93	(0.79–1.09)	0.94	(0.80–1.10)	1.03	(0.89–1.18)	1.03	(0.90–1.18)
Mother’s age (in years)	1.06***	(1.05–1.07)	1.06***	(1.05–1.07)	1.06***	(1.04–1.07)	1.06***	(1.04–1.07)	1.07***	(1.06–1.08)	1.07***	(1.06–1.08)
No. of living children	0.56***	(0.53–0.59)	0.56***	(0.53–0.59)	0.59***	(0.55–0.62)	0.58***	(0.55–0.62)	0.56***	(0.53–0.59)	0.56***	(0.53–0.59)
Mother is employed (ref = No)	1.18**	(1.05–1.32)	1.17**	(1.05–1.31)	1.11	(0.98–1.25)	1.10	(0.98–1.24)	1.04	(0.93–1.16)	1.03	(0.93–1.15)
Source of drinking water												
Improved (ref = unimproved)	0.95	(0.83–1.08)	0.96	(0.84–1.09)	0.89	(0.79–1.00)	0.88*	(0.79–0.99)	0.97	(0.87–1.09)	0.98	(0.88–1.09)
Distance to a medical facility												
Not a problem (ref = problem)	1.03	(0.92–1.15)	1.01	(0.90–1.13)	1.15*	(1.02–1.29)	1.14*	(1.01–1.28)	0.98	(0.87–1.09)	0.97	(0.86–1.08)
Household wealth index	0.90*	(0.81–0.99)	1.07	(0.93–1.22)	0.94	(0.85–1.04)	1.02	(0.89–1.16)	0.89**	(0.82–0.96)	0.93	(0.83–1.04)
Christian (ref = Muslim)	0.97	(0.83–1.14)	1.00	(0.85–1.17)	0.85	(0.71–1.01)	0.86	(0.73–1.03)	1.14	(0.97–1.35)	1.17	(0.99–1.39)
Geopolitical zone (ref = NC)												
Northeast	1.20*	(1.01–1.44)	1.23*	(1.03–1.47)	1.28*	(1.05–1.57)	1.31**	(1.08–1.60)	1.15	(0.97–1.36)	1.16	(0.98–1.37)
Northwest	1.15	(0.96–1.38)	1.17	(0.98–1.41)	1.38**	(1.14–1.68)	1.42***	(1.17–1.72)	1.19*	(1.01–1.41)	1.20*	(1.02–1.42)
Southeast	1.17	(0.93–1.47)	1.16	(0.93–1.46)	1.49***	(1.18–1.88)	1.50***	(1.19–1.91)	0.90	(0.71–1.14)	0.92	(0.72–1.18)
Southsouth	1.11	(0.89–1.40)	1.11	(0.89–1.40)	0.95	(0.74–1.22)	0.95	(0.75–1.22)	0.87	(0.69–1.11)	0.89	(0.70–1.14)
Southwest	0.77*	(0.61–0.96)	0.77*	(0.61–0.97)	0.86	(0.67–1.10)	0.88	(0.68–1.12)	0.69**	(0.55–0.86)	0.71**	(0.57–0.89)
Rural (ref = urban)	1.16	(0.98–1.37)	1.16	(0.99–1.36)	1.17	(1.00–1.37)	1.16	(1.00–1.36)	1.03	(0.89–1.19)	1.03	(0.90–1.19)
EAM # Wealth index												
Homogamy Low # wealth (ref)			1.00	(1.00–1.00)			1.00	(1.00–1.00)			1.00	(1.00–1.00)
Homogamy high # wealth index			0.70***	(0.59–0.84)			0.80*	(0.67–0.97)			0.82**	(0.70–0.95)
Hypergamy # wealth index			0.81*	(0.67–0.99)			0.98	(0.79–1.21)			1.17	(0.95–1.44)
Hypogamy # wealth index			0.78	(0.59–1.03)			0.88	(0.65–1.21)			1.03	(0.78–1.36)
Wald test			Chi (3) =15.98 ** 20,557				Chi (3) = 5.81 22,892				Chi (3) =15.22	
Observations	20,557				22,892				25,827		25,827	

Analyses are clustered at the household level

CI = Confidence Interval

ref. = Reference Group

Model 1 adjusted for covariates, Model 2 added interaction term (EAM#wealth)

(1) Homogamy low –both parents have at most primary school education

(2) Homogamy high –both parents have at least secondary education

(3) Hypergamy –the father has at least secondary and the mother has at most primary

(4) Hypogamy –the father has at most primary and the mother has at least secondary

*** *p* < 0.001, ** *p* < 0.01, * *p* < 0.05

When hypergamy is taken as the reference group and compared to hypogamy, children of mothers in hypogamy had 11% (HR = 0.89; CI = 0.69–1.15; *p* > 0.1) less likelihood of infant mortality, though the result was not statistically significant at *p* < 0.05 (see supplemental Table 2A). These observed results were similar to those of the 2013 and 2008 survey data, but with little variation in the magnitude of the association.

In Model 2, the moderating role of family wealth on the association between EAM and infant mortality was significant in the 2008 and 2013 surveys, but not in 2018. However, the result of the Wald test for the significance of the interaction suggested that the inclusion was only significant in the 2008 survey.

#### The relationship between EAM and child mortality

In the adjusted result of the 2018 survey, children of mothers in high-education homogamy had a 57% (HR = 0.43; CI = 0.32–0.58; *p* < 0.001) lower likelihood of child mortality compared to children of mothers in low-education homogamy (see Table 3, Model 1). Also, compared to children of mothers in low-education homogamy, the likelihood of child mortality was lessened by 27% (HR = 0.73; CI = 0.59–0.91; *p* < 0.05) for children of mothers in hypergamy and also by 27% (HR = 0.73; CI = 0.46–1.14; *p* > 0.1) for children of mothers in hypogamy (see Table 3, Model 1).

**Table 3:** Cox proportional regression showing the adjusted hazard ratio between educational assortative mating, child mortality: 2008–2018 Nigeria DHS

VARIABLES	2008				2013				2018			
	Mode 1		Model 2		Model 1		Model 2		Model 1		Model 2	
	HR	CI	HR	CI	HR	CI	HR	CI	HR	CI	HR	CI
EAM												
Homogamy low (ref)												
Homogamy high	0.66*	(0.47–0.94)	0.75	(0.54–1.04)	0.59**	(0.41–0.85)	0.58**	(0.40–0.84)	0.43***	(0.32–0.58)	0.43***	(0.32–0.57)
Hypergamy	1.13	(0.91–1.41)	1.05	(0.82–1.34)	1.08	(0.84–1.38)	0.97	(0.72–1.32)	0.73**	(0.59–0.91)	0.66**	(0.52–0.85)
Hypogamy	0.83	(0.55–1.26)	0.67	(0.41–1.08)	0.69	(0.41–1.15)	0.64	(0.38–1.08)	0.73	(0.46–1.14)	0.66	(0.42–1.03)
Female (ref = male)	0.93	(0.81–1.07)	0.93	(0.81–1.07)	0.92	(0.78–1.08)	0.92	(0.78–1.08)	0.99	(0.86–1.14)	0.99	(0.86–1.14)
Birth order												
Firstborn (ref)												
2-4th	1.40**	(1.09–1.81)	1.40**	(1.09–1.80)	1.14	(0.86–1.50)	1.13	(0.86–1.50)	1.31*	(1.03–1.66)	1.31*	(1.03–1.66)
5th+	3.86***	(2.88–5.17)	3.82***	(2.85–5.11)	3.08***	(2.25–4.21)	3.07***	(2.24–4.20)	3.93***	(2.97–5.19)	3.90***	(2.95–5.15)
Birth interval												
< 24 months (ref)												
> 24 months	0.62***	(0.53–0.74)	0.62***	(0.53–0.74)	0.57***	(0.47–0.69)	0.57***	(0.47–0.69)	0.59***	(0.50–0.69)	0.59***	(0.50–0.69)
Perceived birth weight												
Small (ref)												
Average	0.82	(0.67–1.01)	0.82	(0.67–1.01)	0.96	(0.77–1.21)	0.96	(0.77–1.20)	1.13	(0.92–1.38)	1.13	(0.92–1.38)
Large	0.79*	(0.64–0.97)	0.78*	(0.64–0.96)	0.84	(0.67–1.05)	0.83	(0.66–1.04)	0.97	(0.78–1.20)	0.97	(0.78–1.20)
Multiple births (ref = singleton)	2.95***	(2.06–4.21)	2.92***	(2.04–4.16)	2.34***	(1.47–3.73)	2.33***	(1.47–3.71)	3.06***	(2.23–4.20)	3.06***	(2.23–4.19)
Place of delivery												
Medical (ref non-medical)	0.82	(0.65–1.04)	0.83	(0.66–1.05)	0.89	(0.68–1.16)	0.89	(0.69–1.16)	0.83	(0.67–1.04)	0.84	(0.68–1.05)
Mother’s age (in years)	1.05***	(1.04–1.07)	1.06***	(1.04–1.07)	1.05***	(1.04–1.07)	1.05***	(1.04–1.07)	1.07***	(1.06–1.08)	1.07***	(1.06–1.08)
No. of living children	0.54***	(0.51–0.59)	0.54***	(0.51–0.59)	0.53***	(0.49–0.58)	0.53***	(0.49–0.58)	0.50***	(0.47–0.54)	0.50***	(0.47–0.54)
Mother is employed (ref = No)	1.04	(0.89–1.21)	1.03	(0.88–1.21)	1.22*	(1.01–1.46)	1.21*	(1.01–1.45)	1.10	(0.95–1.27)	1.09	(0.94–1.26)
Source of drinking water												
Improved (ref = unimproved)	1.03	(0.86–1.23)	1.03	(0.86–1.23)	0.97	(0.81–1.15)	0.97	(0.81–1.15)	0.99	(0.85–1.15)	0.99	(0.86–1.15)
Distance to a medical facility												
Not a problem (ref = problem)	1.10	(0.94–1.29)	1.08	(0.92–1.27)	1.20*	(1.00–1.43)	1.19	(1.00–1.42)	1.10	(0.94–1.28)	1.08	(0.93–1.26)
Household wealth index	0.89	(0.77–1.03)	0.99	(0.82–1.19)	0.79**	(0.68–0.92)	0.85	(0.71–1.03)	0.84**	(0.74–0.94)	0.91	(0.80–1.04)
Christian (ref = Muslim)	1.30*	(1.01–1.67)	1.34*	(1.04–1.73)	1.79***	(1.28–2.51)	1.83***	(1.31–2.56)	1.30	(0.99–1.70)	1.35*	(1.03–1.76)
Geopolitical zone (ref = NC)												
Northeast	1.31	(0.99–1.73)	1.32	(1.00–1.74)	1.51*	(1.10–2.08)	1.52**	(1.11–2.09)	1.11	(0.85–1.45)	1.11	(0.85–1.45)
Northwest	1.54**	(1.17–2.03)	1.56**	(1.18–2.05)	1.43*	(1.04–1.96)	1.44*	(1.05–1.98)	2.05***	(1.61–2.62)	2.06***	(1.62–2.63)
Southeast	1.58*	(1.10–2.27)	1.56*	(1.09–2.25)	2.38***	(1.45–3.91)	2.41***	(1.47–3.95)	0.92	(0.59–1.43)	0.96	(0.61–1.51)
Southsouth	1.31	(0.92–1.87)	1.31	(0.92–1.88)	1.95**	(1.23–3.10)	1.98**	(1.24–3.15)	0.74	(0.43–1.27)	0.77	(0.45–1.32)
Southwest	0.61*	(0.41–0.91)	0.61*	(0.41–0.91)	0.80	(0.49–1.32)	0.82	(0.50–1.35)	0.52**	(0.32–0.84)	0.54*	(0.34–0.87)
Rural (ref = urban)	1.24	(0.97–1.58)	1.24	(0.97–1.57)	1.55**	(1.19–2.03)	1.54**	(1.18–2.01)	1.01	(0.82–1.24)	1.00	(0.81–1.23)
EAM # Wealth index												
Homogamy Low # wealth (ref)			1.00	(1.00–1.00)			1.00	(1.00–1.00)			1.00	(1.00–1.00)
Homogamy high # wealth index			0.68**	(0.51–0.90)			0.84	(0.60–1.17)			0.75*	(0.59–0.95)
Hypergamy # wealth index			0.85	(0.65–1.12)			0.85	(0.61–1.19)			0.86	(0.65–1.13)
Hypogamy # wealth index			1.30	(0.85–1.97)			0.85	(0.44–1.65)			0.78	(0.47–1.30)
Wald test			Chi (3) =11.09* 14,984				Chi (3) =1.64 16,962				Chi (3) =6.33	
Observations	14,984				16,962				19,318		19,318	

Analyses are clustered at the household level

CI = Confidence Interval

ref. = Reference Group

Model 1 adjusted for covariates, Model 2 added interaction term (EAM#wealth)

(1) Homogamy low –both parents have at most primary school education

(2) Homogamy high –both parents have at least secondary education

(3) Hypergamy –the father has at least secondary and the mother has at most primary

(4) Hypogamy –the father has at most primary and the mother has at least secondary

*** *p* < 0.001, ** *p* < 0.01, * *p* < 0.05

When hypergamy is taken as the reference group and compared to hypogamy, children of mothers in hypogamy showed an indication of 1% (HR = 0.99; CI = 0.62–1.58; *p* > 0.1) less likelihood of child mortality, but the result was also not statistically significant, similar to infant mortality (see supplemental Table 2B). These results were also similar to those of the 2013 and 2008 surveys, but with little variation in the magnitude of the association.

The moderating role of family wealth on the association between EAM and child mortality was statistically significant in the 2013 and 2018 data, but not for 2008. The Wald test, however, suggested that the addition of the interaction did not contribute significantly to the explanatory power of the 2018 and 2013 models, but it did for the 2008 model.

Socio-demographic factors associated with the risk of both infant and child mortality include higher birth order, multiple pregnancies (birth), and increasing mother age. On the other hand, factors such as female gender, longer birth intervals, higher birthweight, and higher parity indicate protectiveness.

#### Robustness check

The results of the sensitivity analyses where EAM was re-segmented into at most secondary vs. tertiary showed a near-perfect similarity with the main analysis (see supplemental Table 3). This indicates that how low or high education has been defined did not affect the results. Additionally, the analysis that excluded partners with the same level of education and had EAM conceptualized as a father having any level of education higher than the mother (hypergamy) or a mother having any level of education higher than the father (hypogamy) is presented in the supplemental Table 4. The results were consistent, indicating that how we had conceptualized hypergamy and hypogamy variables in the analysis did not affect the results. Finally, the analysis that excluded those with no years of formal education, and then creating an indicator for whether the number of years of education completed by the mother is lower, equal, or higher than that completed by the father is presented in the supplemental Table 5. It shows that, except for infant mortality in 2008, both infant and child mortalities are lower in hypogamy than in hypergamy when compared to where both couples have the same years of educational attainment. This again showed consistency with the results of our main analysis.

### Estimates of mortality rates

**Fig. 2:**
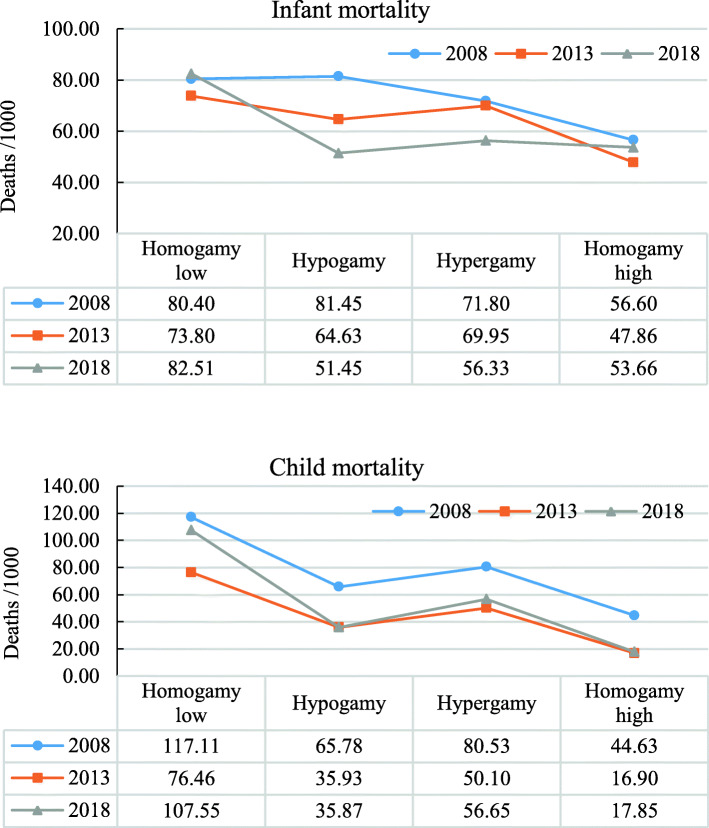
Infant and child mortality rates by groups of educational assortative mating: 2008–2018 Nigeria DHS. (1) Homogamy low –both parents have at most primary school education, (2) Homogamy high –both parents have at least secondary education, (3) Hypergamy –father has at least secondary and the mother has at most primary, (4) Hypogamy – the father has at most primary and the mother has at least secondary

The trend of infant mortality rate per 1000 live births, and child mortality rate per 1000 who survive up to the first year of birth across the spectrum of EAM are presented in Fig. 2. The data shows that, except for infant mortality in 2008, mortality rates, including child mortality, were highest in low-education homogamy. The trend however declined between 2008 and 2013 only to rise again in 2018. Expectedly, both infant and child mortality was lowest in high-education homogamy but the trend over time was also similar to low-education homogamy, declining between 2008 to 2013 and rising again in 2018. We also observed that both infant and child mortality rates were relatively lower where mothers were in hypogamy compared to hypergamous unions, except for infant mortality in 2008. This further echoed the pattern observed in the multivariate analysis.

## Discussion

In this study, we used data obtained from three waves of the Nigeria DHS (2008, 2013, and 2018) to examine trends in the association between EAM and childhood mortality in the context of Nigeria. The Cox proportional hazard regression was employed for multivariate analyses, while estimates of mortality rates across the spectrum of EAM were obtained through the synthetic cohort technique. The findings showed that 1) compared to children of couples in low-education homogamy, the risk of both infant and child mortality is lower in any other combination of EAM, and particularly lowest among those of high-education homogamy. 2) Compared to children of mothers in hypergamous unions, those of mothers in hypogamous unions had a seemingly lower likelihood of both infant and child mortality, although not statistically significant. These findings were consistent across the three waves.

Furthermore, the mortality rates were mostly highest in low-education homogamy, lowest in high-education homogamy, and lower in hypogamy compared to hypergamy. While these mortality trends decreased between 2008 and 2018, they were higher in 2018 than in 2013. The current study is significant because it suggests that, in addition to the absolute level of education, the similarity of parents’ education may be related to child survival, alluding to the family system theory.

The finding is partly consistent with another study from SSA [7] but different in part from the 21-cross-country study of SSA [23]. This could be subject to a difference in the sample of analysis. While the 21-country study looked at under-5 children as a whole, our study looked at infant and child mortality separately.

In the current study, high-education homogamy had a strong association with reduced infant and child mortality, while low-education homogamy had the highest risk of childhood mortality. Parents who both have high education are more likely to understand the importance of maternal and child healthcare (MCH), thereby more likely to have a consensus in its utilization. Forms of MCH such as antenatal care or birth delivery in a health facility are associated with reduced childhood mortality [26, 42]. Also, in regards to genotype compatibility, medical evidence has suggested that two sickle cell carriers (AS, SS) are least compatible (at least to the extent that such a union hopes to produce offspring) [43]. The enlightenment gained from education could help bring this to realization, thus, leading prospective couples to avoid forming a union where it is possible to have a child with a sickle cell disease, as they are more susceptible to mortality [44, 45].

Secondly, improved education could translate into better job opportunities and, by implication, better financial income, thereby offering affordability for medical costs or insurance. By contrast, low education attainment may be attributed to poor household income, accompanied by poor nutritional intake, making infants and young children more susceptible to sickness [35, 46].

In the multivariate, sensitivity, and mortality rates analyses, childhood mortality showed a lower likelihood in hypogamy compared to hypergamy, consistent with similar studies [47, 48]. Women’s education has been widely reported to be important for child survival, as a result of their improved agency [49–52]. Though the husband’s education is also important, such that it may compensate where the mother’s education falls short, but it will depend on the quality of support on the part of the man. Furthermore, this phenomenon may also be explained from two theoretical perspectives, the second complementing the first. First, is the theory of parental investment [53], postulating that an individual could contribute to reproductive effort either through “mating” or “parenting” (offspring quantity vs. offspring quality). It posits that males tend to favor investment in mating (quantity), while females favor investment in parenting (quality). The second is the theory of maternal altruism [54], which extends from human psychology. It stipulates that women, by the virtue of their identities as mothers and wives, are “naturally” predisposed towards nurturing and self-sacrifice. This could imply that mothers are more likely than fathers to mobilize their resources, both material and non-material, to better care for their children.

The findings from this study reiterate the fact that parents’ education is important for infant and child survival. Therefore, policy measures should include promoting a policy of “education for all” and, particularly, female education, which is already in tandem with the 4th agenda of sustainable development goals (SDGs). While women’s education in Nigeria has attracted some policy attention and some marked improvements have been observed over the years, regional variation still exists. As also indicated by the results in the geopolitical zone in the multivariate analysis, regional-specific policies may be more relevant. In regards to child health outcomes, the mother’s education may likely compensate where the father’s education falls short.

Secondly, in addition to women’s empowerment, paternal involvement in prenatal care should also be used as a strategy for better feto-natal health outcomes. Paternal involvement is associated with better prenatal care [55], and the quality of prenatal care associated with feto-infant health and survival [56]. Third, relevant agencies should promote campaigns educating people with low educational attainment on the most appropriate and cost-effective child-care practices to reduce both infant and child mortality among this population group.

Additionally, most studies on educational assortative mating have mostly considered homogamy as a single construct without further dichotomization into low or high education. However, the findings in this study show that significant differences exist between both, particularly regarding child health outcomes. Therefore, the findings in this study may offer some implications for theory.

### Limitation and strength

The limitations associated with this study include the use of cross-sectional data, which does not allow for the inference of a causal relationship. Second, there may be instances of under-reporting of mortality due to cultural reasons that forbid speaking of the dead or the emotional pain associated with recalling a lost child. Third, due to the few samples of neonatal mortality, we analyzed neonatal mortality together with infant mortality. Lastly, the educational attainment of the father was as reported by the mother. There is the possibility of error, particularly in the sensitivity analysis in supplemental Table 5, where we focused on years of education completed. Despite the limitations, the strength of the study lies in the use of large and nationally representative data, and the use of a frailty model to account for the correlation between samples at the household level.

## Conclusion

This study has shown that beyond the absolute level of education, the similarity of parental education, conceptualized as EAM, is associated with child survival, alluding to the family system theory. Findings from this study have social, health, and theoretical implications. First, basic and compulsory educational attainment should be made a statutory provision and freely accessible. Secondly, campaigns relating to both reproductive and pediatric health should be targeted at the population group most susceptible to infant and child mortality, and content should be regional-specific. Third, since education is relevant to health behaviors and by extension, health outcomes, it may be important to conceptualize EAM a bit differently in public health studies than in a pure sociological context. Future studies could investigate how EAM is associated with other aspects of child wellbeing or could show how the magnitude of the association varies when marital status is put into consideration.

## Supplementary Information

**Additional file 1: Figure S1.** Test of proportional hazards assumption for EAM.

**Additional file 2: Table S1.** Cox proportional regression showing the unadjusted hazard ratio between educational assortative mating (EAM), infant and child mortality: 2008-2018 Nigeria DHS.

**Additional file 3: Table S2.** Cox proportional regression comparing the adjusted hazard ratio of infant and child mortality between hypergamy and hypogamy: 2008-2018 Nigeria DHS.

**Additional file 4: Table S3.** Cox proportional regression showing the adjusted hazard ratio between the alternative measure of educational assortative mating, infant and child mortality: 2008-2018 Nigeria DHS.

**Additional file 5: Table S4.** Cox proportional regression showing the adjusted hazard ratio of infant and child mortality between the alternative measure of hypergamy and hypogamy: 2008-2018 Nigeria DHS.

**Additional file 6: Table S5.** Cox proportional regression showing the adjusted hazard ratio between difference in years of education (DYE), infant and child mortality: 2008-2018 Nigeria DHS.

## Data Availability

The datasets used for analysis and reaching the conclusions of this study is available online at MEASURE DHS (https://www.dhsprogram.com/data/available-datasets.cfm). It is released upon request subject to approval.

## Abbreviations

DHS: Demographic and Health Survey
EAM: Educational Assortative Mating
MCH: Maternal and Child Healthcare
NPC: National Population Commission
PSU: Primary Sampling Unit
SDGs: Social Development Goals
SSA: Sub-Sahara African
