# Supplementary material for: Trends in the association between educational assortative mating, infant and child mortality in Nigeria
Source: BMC Public Health. 2021 Aug 3;21:1493. doi: 10.1186/s12889-021-11568-0 (PMC8330029; doi:10.1186/s12889-021-11568-0)
Supplement: Supplementary file 1 — Additional file 1: Figure S1. Test of proportional hazards assumption for EAM. [file 12889_2021_11568_MOESM1_ESM.docx]

Supplemental Figure 1: Test of proportional hazards assumption for EAM

(1) Homogamy low –both parents have at most primary school education,

(2) Homogamy high –both parents have at least secondary education,

(3) Hypergamy –father has at least secondary and the mother has at most primary,

(4) Hypogamy – the father has at most primary and the mother has at least secondary
