# Supplementary material for: Trends in the association between educational assortative mating, infant and child mortality in Nigeria
Source: BMC Public Health. 2021 Aug 3;21:1493. doi: 10.1186/s12889-021-11568-0 (PMC8330029; doi:10.1186/s12889-021-11568-0)
Supplement: Supplementary file 2 — Additional file 2: Table S1. Cox proportional regression showing the unadjusted hazard ratio between educational assortative mating (EAM), infant and child mortality: 2008-2018 Nigeria DHS. [file 12889_2021_11568_MOESM2_ESM.docx]

| Supplemental Table 1 | | | | | | |
| --- | --- | --- | --- | --- | --- | --- |
| A: Cox proportional regression showing the unadjusted hazard ratio between educational assortative mating (EAM)  and infant mortality: 2008-2018 Nigeria DHS | | | | | | |
|  | 2008 | | 2013 | | 2018 | |
| VARIABLES | HR | CI | HR | CI | HR | CI |
| EAM |  |  |  |  |  |  |
| Homogamy high (ref= low) | 0.69*** | (0.60-0.80) | 0.64*** | (0.55-0.73) | 0.68*** | (0.60-0.77) |
| Hypogamy (ref= Hypergamy) | 1.08 | (0.82-1.41) | 1.04 | (0.80-1.36) | 1.13 | (0.85-1.48) |
|  |  |  |  |  |  |  |
| Observations | 21445 | | 23802 | | 26476 | |
|  |  |  |  |  |  |  |
|  |  |  |  |  |  |  |
| B: Cox proportional regression showing the unadjusted hazard ratio between educational assortative mating (EAM)  and child mortality: 2008-2018 Nigeria DHS | | | | | | |
|  | 2008 | | 2013 | | 2018 | |
| VARIABLES | HR | CI | HR | CI | HR | CI |
| EAM |  |  |  |  |  |  |
| Homogamy high (ref= low) | 0.39*** | (0.30-0.50) | 0.27*** | (0.20-0.35) | 0.21*** | (0.17-0.27) |
| Hypogamy (ref= Hypergamy) | 0.67 | (0.45-1.01) | 0.59* | (0.36-0.98) | 0.82 | (0.51-1.30) |
|  |  |  |  |  |  |  |
| Observations | 15604 | | 17629 | | 19773 | |
| 1. Analyses are clustered at the household level 2. CI= Confidence Interval 3. *** p<0.001, **p<0.01, * p<0.05 4. ref= Reference Group 5. (1) Homogamy low –both parents have at most primary school education,   (2) Homogamy high –both parents have at least secondary education,  (3) Hypergamy –the father has at least secondary and the mother has at most primary,  (4) Hypogamy –the father has at most primary and the mother has at least secondary. | | | | | | |
