# Supplementary material for: Trends in the association between educational assortative mating, infant and child mortality in Nigeria
Source: BMC Public Health. 2021 Aug 3;21:1493. doi: 10.1186/s12889-021-11568-0 (PMC8330029; doi:10.1186/s12889-021-11568-0)
Supplement: Supplementary file 3 — Additional file 3: Table S2. Cox proportional regression comparing the adjusted hazard ratio of infant and child mortality between hypergamy and hypogamy: 2008-2018 Nigeria DHS. [file 12889_2021_11568_MOESM3_ESM.docx]

| Supplemental Table 2 | | | | | | | | | | | | |
| --- | --- | --- | --- | --- | --- | --- | --- | --- | --- | --- | --- | --- |
| A: Cox proportional regression comparing the adjusted hazard ratio of infant mortality between hypergamy and hypogamy: 2008-2018 Nigeria DHS | | | | | | | | | | | | |
|  | 2008 | | | | 2013 | | | | 2018 | | | |
|  | Mode 1 | | Model 2 | | Model 1 | | Model 2 | | Model 1 | | Model 2 | |
| VARIABLES | HR | CI | HR | CI | HR | CI | HR | CI | HR | CI | HR | CI |
| EAM |  |  |  |  |  |  |  |  |  |  |  |  |
| Hypogamy (ref= Hypergamy) | 0.80 | (0.62-1.05) | 0.83 | (0.64-1.08) | 0.88 | (0.67-1.16) | 0.88 | (0.67-1.16) | 0.89 | (0.69-1.15) | 0.85 | (0.66-1.10) |
|  |  |  |  |  |  |  |  |  |  |  |  |  |
| EAM # Wealth index |  |  |  |  |  |  |  |  |  |  |  |  |
| Hypogamy # wealth (ref= hypergamy) |  |  | 0.96 | (0.71-1.29) |  |  | 0.90 | (0.64-1.27) |  |  | 0.88 | (0.65-1.20) |
|  |  |  |  |  |  |  |  |  |  |  |  |  |
| Wald test |  |  | Chi (3) =15.98** | |  |  | Chi (3) =5.81 | |  |  | Chi (3) =15.22** | |
| Observations |  | 20557 | 20557 | | 22892 |  | 22892 | | 25827 | | 25827 | |
|  |  |  |  |  |  |  |  |  |  |  |  |  |
|  |  |  |  |  |  |  |  |  |  |  |  |  |
|  |  |  |  |  |  |  |  |  |  |  |  |  |
| B: Cox proportional regression comparing the adjusted hazard ratio of child mortality between hypergamy and hypogamy: 2008-2018 Nigeria DHS | | | | | | | | | | | | |
|  | 2008 | | | | 2013 | | | | 2018 | | | |
|  | Mode 1 | | Model 2 | | Mode 1 | | Model 2 | | Mode 1 | | Model 2 | |
| VARIABLES | HR | CI | HR | CI | HR | CI | HR | CI | HR | CI | HR | CI |
| EAM |  |  |  |  |  |  |  |  |  |  |  |  |
| Hypogamy (ref= Hypergamy) | 0.73 | (0.47-1.12) | 0.64 | (0.39-1.04) | 0.64 | (0.38-1.08) | 0.66 | (0.39-1.13) | 0.99 | (0.62-1.58) | 0.99 | (0.61-1.61) |
|  |  |  |  |  |  |  |  |  |  |  |  |  |
| EAM # Wealth index |  |  |  |  |  |  |  |  |  |  |  |  |
| Hypogamy # wealth (ref= hypergamy) |  |  | 1.52 | (0.98-2.37) |  |  | 1.00 | (0.50-2.02) |  |  | 0.91 | (0.52-1.59) |
|  |  |  |  |  |  |  |  |  |  |  |  |  |
| Wald test |  |  | Chi (3) =11.09*  14,984 | |  |  | Chi (3) =1.64  16962 | |  |  | Chi (3) =6.33 | |
| Observations | 14984 | |  |  | 16962 | |  |  | 19318 | | 19318 | |
| 1. Analyses are clustered at the household level 2. CI= Confidence Interval 3. *** p<0.001, **p<0.01, * p<0.05 4. ref= Reference Group 5. Model adjusted for covariates, Model 2 Added interaction term (EAM # wealth)   (1) Hypergamy –the father has at least secondary and the mother has at most primary,  (2) Hypogamy –the father has at most primary and the mother has at least secondary. | | | | | | | | | | | | |
