# Supplementary material for: Trends in the association between educational assortative mating, infant and child mortality in Nigeria
Source: BMC Public Health. 2021 Aug 3;21:1493. doi: 10.1186/s12889-021-11568-0 (PMC8330029; doi:10.1186/s12889-021-11568-0)
Supplement: Supplementary file 4 — Additional file 4: Table S3. Cox proportional regression showing the adjusted hazard ratio between the alternative measure of educational assortative mating, infant and child mortality: 2008-2018 Nigeria DHS. [file 12889_2021_11568_MOESM4_ESM.docx]

| Supplemental Table 3 | | | | | | | | | | | | |
| --- | --- | --- | --- | --- | --- | --- | --- | --- | --- | --- | --- | --- |
| A: Cox proportional regression showing the adjusted hazard ratio between the alternative measure of educational assortative mating, infant mortality: 2008-2018 Nigeria DHS | | | | | | | | | | | | |
|  | 2008 | | | | 2013 | | | | 2018 | | | |
|  | Mode 1 | | Model 2 | | Model 1 | | Model 2 | | Model 1 | | Model 2 | |
| VARIABLES | HR | CI | HR | CI | HR | CI | HR | CI | HR | CI | HR | CI |
| EAM |  |  |  |  |  |  |  |  |  |  |  |  |
| Homogamy low (ref) |  |  |  |  |  |  |  |  |  |  |  |  |
| Homogamy high | 0.43*** | (0.27-0.67) | 0.67 | (0.29-1.57) | 0.65* | (0.46-0.91) | 0.47* | (0.22-0.98) | 0.56*** | (0.41-0.77) | 0.56* | (0.33-0.95) |
| Hypergamy | 0.81 | (0.63-1.04) | 0.80 | (0.61-1.05) | 0.84 | (0.67-1.05) | 0.82 | (0.65-1.05) | 0.90 | (0.74-1.09) | 0.93 | (0.76-1.14) |
| Hypogamy | 0.56 | (0.30-1.04) | 1.22 | (0.53-2.83) | 0.47* | (0.26-0.84) | 0.85 | (0.44-1.65) | 0.86 | (0.61-1.21) | 0.65 | (0.31-1.37) |
|  |  |  |  |  |  |  |  |  |  |  |  |  |
| EAM # Wealth index |  |  |  |  |  |  |  |  |  |  |  |  |
| Homogamy low # wealth (ref) |  |  | 1.00 | (1.00-1.00) |  |  | 1.00 | (1.00-1.00) |  |  | 1.00 | (1.00-1.00) |
| Homogamy high # wealth index |  |  | 0.73 | (0.43-1.24) |  |  | 1.26 | (0.80-1.98) |  |  | 1.00 | (0.72-1.39) |
| Hypergamy # wealth index |  |  | 1.00 | (0.79-1.26) |  |  | 1.06 | (0.80-1.40) |  |  | 0.90 | (0.72-1.12) |
| Hypogamy # wealth index |  |  | 0.47 | (0.22-1.04) |  |  | 0.54* | (0.31-0.92) |  |  | 1.31 | (0.73-2.35) |
|  |  |  |  |  |  |  |  |  |  |  |  |  |
| Wald test |  |  | Chi (3) =4.73 | |  |  | Chi (3) =6.43 | |  |  | Chi (3) =1.78 | |
| Observations |  | 20557 | 20557 | | 22892 |  | 22892 | | 25827 | | 25827 | |
|  |  |  |  |  |  |  |  |  |  |  |  |  |
|  |  |  |  |  |  |  |  |  |  |  |  |  |
|  |  |  |  |  |  |  |  |  |  |  |  |  |
| B: Cox proportional regression showing the adjusted hazard ratio between the alternative measure of educational assortative mating, child mortality: 2008-2018 Nigeria DHS | | | | | | | | | | | | |
|  | 2008 | | | | 2013 | | | | 2018 | | | |
|  | Mode 1 | | Model 2 | | Mode 1 | | Model 2 | | Mode 1 | | Model 2 | |
| VARIABLES | HR | CI | HR | CI | HR | CI | HR | CI | HR | CI | HR | CI |
| EAM |  |  |  |  |  |  |  |  |  |  |  |  |
| Homogamy low (ref) |  |  |  |  |  |  |  |  |  |  |  |  |
| Homogamy high | 0.11** | (0.03-0.47) | 0.23 | (0.05-1.11) | 0.31* | (0.11-0.84) | 0.20 | (0.04-1.12) | 0.48* | (0.26-0.87) | 0.49 | (0.16-1.46) |
| Hypergamy | 0.93 | (0.66-1.29) | 0.90 | (0.63-1.29) | 0.62* | (0.39-0.98) | 0.64 | (0.41-1.01) | 0.46*** | (0.30-0.71) | 0.48*** | (0.32-0.74) |
| Hypogamy | 0.31 | (0.08-1.17) | 0.07 | (0.00-2.59) | 0.00*** | (0.00-0.00) | 0.00*** | (0.00-0.00) | 0.43 | (0.16-1.17) | 0.70 | (0.26-1.91) |
|  |  |  |  |  |  |  |  |  |  |  |  |  |
| EAM # Wealth index |  |  |  |  |  |  |  |  |  |  |  |  |
| Homogamy Low # wealth (ref) |  |  | 1.00 | (1.00-1.00) |  |  | 1.00 | (1.00-1.00) |  |  | 1.00 | (1.00-1.00) |
| Homogamy high # wealth index |  |  | 0.63 | (0.36-1.11) |  |  | 1.35 | (0.50-3.62) |  |  | 0.96 | (0.43-2.16) |
| Hypergamy # wealth index |  |  | 1.08 | (0.80-1.46) |  |  | 0.76 | (0.47-1.24) |  |  | 0.78 | (0.50-1.23) |
| Hypogamy # wealth index |  |  | 2.81 | (0.33-23.95) |  |  | 1.65*** | (1.25-2.18) |  |  | 0.35*** | (0.23-0.55) |
|  |  |  |  |  |  |  |  |  |  |  |  |  |
| Wald test |  |  | Chi (3) =3.80  14,984 | |  |  | Chi (3) =15.22**  16962 | |  |  | Chi (3) =22.40*** | |
| Observations | 14984 | |  |  | 16962 | |  |  | 19318 | | 19318 | |
| 1. Analyses are clustered at the household level 2. CI= Confidence Interval 3. *** p<0.001, **p<0.01, * p<0.05 4. ref= Reference Group 5. Model adjusted for covariates, Model 2 Added interaction term (EAM # wealth) 6. (1) Homogamy low –both parents have at most secondary school education,   (2) Homogamy high –both parents have at least tertiary education,  (3) Hypergamy –the father has at least tertiary and the mother has at most secondary,  (4) Hypogamy –the father has at most secondary and the mother has at least tertiary. | | | | | | | | | | | | |
