# Supplementary material for: Trends in the association between educational assortative mating, infant and child mortality in Nigeria
Source: BMC Public Health. 2021 Aug 3;21:1493. doi: 10.1186/s12889-021-11568-0 (PMC8330029; doi:10.1186/s12889-021-11568-0)
Supplement: Supplementary file 5 — Additional file 5: Table S4. Cox proportional regression showing the adjusted hazard ratio of infant and child mortality between the alternative measure of hypergamy and hypogamy: 2008-2018 Nigeria DHS. [file 12889_2021_11568_MOESM5_ESM.docx]

| Supplemental Table 4 | | | | | | | | | | | | |
| --- | --- | --- | --- | --- | --- | --- | --- | --- | --- | --- | --- | --- |
| A: Cox proportional regression showing the adjusted hazard ratio of infant mortality between the alternative measure of hypergamy and hypogamy: 2008-2018 Nigeria DHS | | | | | | | | | | | | |
|  | | | | | | | | | | | | |
|  | 2008 | | | | 2013 | | | | 2018 | | | |
|  | Mode 1 | | Model 2 | | Model 1 | | Model 2 | | Model 1 | | Model 2 | |
| VARIABLES | HR | CI | HR | CI | HR | CI | HR | CI | HR | CI | HR | CI |
| EAM |  |  |  |  |  |  |  |  |  |  |  |  |
| Hypogamy (ref= Hypergamy) | 0.95 | (0.78-1.15) | 0.94 | (0.77-1.15) | 0.93 | (0.75-1.15) | 0.91 | (0.74-1.12) | 1.08 | (0.89-1.31) | 1.08 | (0.89-1.31) |
|  |  |  |  |  |  |  |  |  |  |  |  |  |
| EAM # Wealth index |  |  |  |  |  |  |  |  |  |  |  |  |
| Hypogamy # wealth (ref= hypergamy) |  |  | 0.96 | (0.79-1.18) |  |  | 0.89 | (0.72-1.10) |  |  | 0.99 | (0.82-1.21) |
|  |  |  |  |  |  |  |  |  |  |  |  |  |
| Wald test |  |  | Chi (1) =0.12 | |  |  | Chi (1) =1.14 | |  |  | Chi (1) =0.00 | |
| Observations |  |  | 7,892 | |  |  | 8,679 | |  |  | 9,875 | |
|  |  |  |  |  |  |  |  |  |  |  |  |  |
|  |  |  |  |  |  |  |  |  |  |  |  |  |
|  |  |  |  |  |  |  |  |  |  |  |  |  |
| B: Cox proportional regression showing the adjusted hazard ratio of child mortality between the alternative measure of hypergamy and hypogamy : 2008-2018 Nigeria DHS | | | | | | | | | | | | |
|  | 2008 | | | | 2013 | | | | 2018 | | | |
|  | Mode 1 | | Model 2 | | Mode 1 | | Model 2 | | Mode 1 | | Model 2 | |
| VARIABLES | HR | CI | HR | CI | HR | CI | HR | CI | HR | CI | HR | CI |
| EAM |  |  |  |  |  |  |  |  |  |  |  |  |
| Hypogamy (ref= Hypergamy) | 0.75 | (0.56-1.02) | 0.76 | (0.56-1.03) | 0.81 | (0.56-1.19) | 0.74 | (0.46-1.17) | 0.99 | (0.71-1.38) | 0.94 | (0.64-1.37) |
|  |  |  |  |  |  |  |  |  |  |  |  |  |
| EAM # Wealth index |  |  |  |  |  |  |  |  |  |  |  |  |
| Hypogamy # wealth (ref= hypergamy) |  |  | 1.05 | (0.77-1.44) |  |  | 0.83 | (0.52-1.33) |  |  | 0.88 | (0.62-1.26) |
|  |  |  |  |  |  |  |  |  |  |  |  |  |
| Wald test |  |  | Chi (1) =0.10  5,771 | |  |  | Chi (1) =0.62  6,429 | |  |  | Chi (1) =0.47 | |
| Observations | 5,771 | |  |  | 6,429 | |  |  | 7,360 | | 7,360 | |
| 1. Analyses are clustered at the household level 2. Model 1 Adjusted for covariates, Model 2 Added interaction term (EAM # wealth) 3. CI= Confidence Interval 4. *** p<0.001, **p<0.01, * p<0.05 5. ref= Reference group 6. Model 1 adjusted for covariates, Model 2 added interaction (EAM #wealth)   (1) Hypergamy –father has a higher level of educational attainment than mother  (2) Hypogamy –mother has a higher level of educational attainment than father | | | | | | | | | | | | |
