# Supplementary material for: Trends in the association between educational assortative mating, infant and child mortality in Nigeria
Source: BMC Public Health. 2021 Aug 3;21:1493. doi: 10.1186/s12889-021-11568-0 (PMC8330029; doi:10.1186/s12889-021-11568-0)
Supplement: Supplementary file 6 — Additional file 6: Table S5. Cox proportional regression showing the adjusted hazard ratio between difference in years of education (DYE), infant and child mortality: 2008-2018 Nigeria DHS. [file 12889_2021_11568_MOESM6_ESM.docx]

| Supplemental Table 5 | | | | | | | | | | | | |
| --- | --- | --- | --- | --- | --- | --- | --- | --- | --- | --- | --- | --- |
| A: Cox proportional regression showing the adjusted hazard ratio between difference in years of education (DYE) and infant mortality:  2008-2018 Nigeria DHS | | | | | | | | | | | | |
|  | 2008 DHS | | | | 2013 DHS | | | | 2018 DHS | | | |
|  | Mode 1 | | Model 2 | | Model 1 | | Model 2 | | Model 1 | | Model 2 | |
| VARIABLES | HR | CI | HR | CI | HR | CI | HR | CI | HR | CI | HR | CI |
| DYE |  |  |  |  |  |  |  |  |  |  |  |  |
| Equal (ref) |  |  |  |  |  |  |  |  |  |  |  |  |
| Lower | 1.03 | (0.81-1.30) | 1.07 | (0.81-1.43) | 1.24 | (0.99-1.55) | 1.27 | (0.97-1.67) | 1.12 | (0.94-1.34) | 1.08 | (0.89-1.29) |
| Higher | 1.04 | (0.71-1.51) | 1.23 | (0.78-1.93) | 0.62* | (0.41-0.94) | 0.72 | (0.43-1.19) | 0.97 | (0.72-1.32) | 0.96 | (0.65-1.41) |
|  |  |  |  |  |  |  |  |  |  |  |  |  |
| DYE # Wealth index |  |  |  |  |  |  |  |  |  |  |  |  |
| Equal # wealth (ref) |  |  |  |  |  |  |  |  |  |  |  |  |
| Lower # wealth |  |  | 0.95 | (0.74-1.21) |  |  | 0.97 | (0.76-1.25) |  |  | 1.11 | (0.93-1.32) |
| Higher # wealth |  |  | 0.80 | (0.54-1.19) |  |  | 0.81 | (0.49-1.34) |  |  | 1.04 | (0.72-1.50) |
|  |  |  |  |  |  |  |  |  |  |  |  |  |
| Wald test |  |  | Chi (2) =1.21 | |  |  | Chi (2) =0.68 | |  |  | Chi (2) =1.46 | |
| Observations |  |  | 7,519 | |  |  | 9,697 | | 12,401 | | 12,401 | |
|  |  |  |  |  |  |  |  |  |  |  |  |  |
|  |  |  |  |  |  |  |  |  |  |  |  |  |
|  |  |  |  |  |  |  |  |  |  |  |  |  |
| B: Cox proportional regression showing the adjusted hazard ratio between difference in years of education (DYE) and child mortality:  2008-2018 Nigeria DHS | | | | | | | | | | | | |
|  | 2008 DHS | | | | 2013 DHS | | | | 2018 DHS | | | |
|  | Mode 1 | | Model 2 | | Mode 1 | | Model 2 | | Mode 1 | | Model 2 | |
| VARIABLES | HR | CI | HR | CI | HR | CI | HR | CI | HR | CI | HR | CI |
| DYE |  |  |  |  |  |  |  |  |  |  |  |  |
| Equal (ref) |  |  |  |  |  |  |  |  |  |  |  |  |
| Lower | 1.38 | (0.87-2.18) | 1.21 | (0.74-2.00) | 1.23 | (0.78-1.95) | 1.47 | (0.83-2.59) | 1.55* | (1.01-2.38) | 1.53 | (0.99-2.36) |
| Higher | 0.66 | (0.26-1.68) | 0.36 | (0.07-1.73) | 1.09 | (0.51-2.31) | 1.61 | (0.72-3.56) | 0.75 | (0.33-1.67) | 0.94 | (0.42-2.13) |
|  |  |  |  |  |  |  |  |  |  |  |  |  |
| DYE # Wealth index |  |  |  |  |  |  |  |  |  |  |  |  |
| Equal # wealth (ref) |  |  |  |  |  |  |  |  |  |  |  |  |
| Lower # wealth |  |  | 1.23 | (0.81-1.87) |  |  | 0.77 | (0.46-1.28) |  |  | 1.11 | (0.79-1.55) |
| Higher # wealth |  |  | 2.02 | (0.63-6.45) |  |  | 0.35 | (0.09-1.36) |  |  | 0.49** | (0.29-0.83) |
|  |  |  |  |  |  |  |  |  |  |  |  |  |
| Wald test |  |  | Chi (2) =1.83  5,456 | |  |  | Chi (2) =2.61  7,162 | |  |  | Chi (2) =10.89** | |
| Observations | 5,456 | |  |  | 7,162 | |  |  | 19,318 | | 9,256 | |
| 1. Analyses are clustered at the household level 2. CI= Confidence Interval 3. *** p<0.001, **p<0.01, * p<0.05 4. ref= Reference group 5. Model 1 Adjusted for covariates, Model 2 Added interaction term (DYE # wealth) 6. (1) Equal- mother has the same years of education as father   (2) Lower- mother has lower years of education than the father  (3) Higher- mother has more years of education than the father | | | | | | | | | | | | |
